# Enhancing Anthocyanin Extraction from Wine Lees: A Comprehensive Ultrasound-Assisted Optimization Study

**DOI:** 10.3390/antiox12122074

**Published:** 2023-12-05

**Authors:** Marcelo A. Umsza-Guez, Mercedes Vázquez-Espinosa, Nuria Chinchilla, María José Aliaño-González, Carolina Oliveira de Souza, Kodjovi Ayena, Gerardo Fernández Barbero, Miguel Palma, Ceferino Carrera

**Affiliations:** 1 Food Science Postgraduate Program, Faculty of Pharmacy, Federal University of Bahia, Salvador 40170-100, Bahia, Brazil; marcelo.umsza@ufba.br (M.A.U.-G.); carolods@ufba.br (C.O.d.S.); kayena@ufba.br (K.A.); 2 Department of Analytical Chemistry, Faculty of Sciences, University of Cadiz, Agrifood Campus of International Excellence (ceiA3), Wine and Agrifood Research Institute (IVAGRO), 11510 Puerto Real, Spain; mercedes.vazquez@uca.es (M.V.-E.); gerardo.fernandez@uca.es (G.F.B.); miguel.palma@uca.es (M.P.); ceferino.carrera@uca.es (C.C.); 3 Department of Organic Chemistry, Faculty of Sciences, University of Cadiz, Institute of Biomolecules (INBIO), 11510 Puerto Real, Spain; nuria.chinchilla@uca.es; 4 MED–Mediterranean Institute for Agriculture, Environment and Development, Faculdade de Ciências e Tecnologia, Campus de Gambelas, Ed. 8, Universidade do Algarve, 8005-139 Faro, Portugal

**Keywords:** wine lees, by-products, ultrasound-assisted extraction, anthocyanins, antioxidant activity, Box–Behnken design, circular economy

## Abstract

Wine lees, an important by-product of the wine industry, pose a major environmental problem due to the enormous quantities of solid–liquid waste that are discarded annually without defined applications. In this study, the optimization of a method based on a Box–Behnken design with surface response has been carried out to obtain extracts with high anthocyanin content and potent antioxidant activity. Six variables have been considered: %EtOH, temperature, amplitude, cycle, pH, and ratio. The developed method exhibited important repeatability properties and intermediate precision, with less than 5% CV being achieved. Furthermore, these novel methods were successfully applied to diverse wine lees samples sourced from Cabernet Sauvignon and Syrah varieties (*Vitis vinifera*), resulting in extracts enriched with significant anthocyanin content and noteworthy antioxidant activity. Additionally, this study evaluated the influence of grape variety, fermentation type (alcoholic or malolactic), and sample treatment on anthocyanin content and antioxidant activity, providing valuable insights for further research and application in various sectors. The potential applications of these high-quality extracts extend beyond the winemaking industry, holding promise for fields like medicine, pharmaceuticals, and nutraceuticals, thus promoting a circular economy and mitigating environmental contamination.

## 1. Introduction

The winemaking industry plays a crucial role in global agriculture and economy, particularly in Southern Europe, where Spain stands out as a significant producer, having contributed 35 million hL of wine in the past year, accounting for 13.8% of the world’s total production [1]. However, this industry also generates substantial quantities of byproducts and residues, such as grape pomace, vine shoots, grape seeds, and wine lees, presenting significant environmental and economic challenges [2].

Numerous studies have examined the potential recovery and valorization of these residues, exploring avenues such as ethanol production, animal feed utilization, biogas generation, and oil extraction [3,4,5,6,7]. However, comparatively little attention has been directed toward strategies for the valorization of wine lees, making them a particularly troublesome residue with unresolved issues [2].

Wine lees are the sedimentary deposits that form during aging and clarification processes, consisting of grape solids, yeast cells, tartrates, and other precipitates [8,9]. Their organic content and potential to pollute water bodies if improperly managed give rise to environmental concerns. Furthermore, the disposal of wine lees incurs substantial costs, underscoring the need for sustainable solutions to address this matter.

Recent research has unveiled the presence of significant concentrations of bioactive compounds in wine lees, including minerals, yeast cell wall polysaccharides such as *β*-glucans and mannoproteins, organic acids, phenolic acids, stilbenes, flavonols, flavanols, and anthocyanins, among others [10,11,12]. 

Anthocyanins represent a category of bioactive compounds from the polyphenols family that have gained widespread recognition for their health-enhancing properties, which include antioxidant, antimicrobial, and anti-inflammatory effects. These attributes have significant implications for the prevention and treatment of various conditions, such as metabolic syndromes, neurodegenerative disorders, cardiovascular diseases, diabetes, and even cancer [13,14,15]. In the context of wine lees, the average content of total polyphenolic compounds has been reported to range from 190 to 1630 mg/g of wine lees, with an important influence of the variety. In addition, average anthocyanins content has been proven to vary from 10 to 31 mg/g in wine lees [16,17,18]. Remarkably, the presence of these compounds in wine lees has been associated with important activities, including antioxidant effects [19] and the modulation of proliferation in sheep peripheral blood mononuclear cells [20]. Numerous extraction techniques have been described to recover polyphenols from wine lees, encompassing solid–liquid extraction using organic solvents, supercritical CO_2_ extraction, enzymatic hydrolysis, microwave-assisted extraction, and membrane technology [21,22,23,24].

Ultrasound-assisted extraction (UAE) has emerged as a highly regarded and efficient method for extracting polyphenols from diverse food products and by-products [25,26]. This technique involves subjecting the sample to ultrasonic waves, generating cavitation bubbles near the cell surface, which results in shock waves that contribute to the disruption of cell wall. This allows for a higher contact area between bioactive compounds and the extractant, promoting mass transfer and improving the extraction efficiency [27]. One of the prominent advantages of UAE lies in its ability to substantially reduce both extraction time and solvent consumption, rendering it an environmentally conscious alternative to conventional extraction methods [28]. Furthermore, UAE has been shown to yield higher amounts of polyphenols compared to traditional techniques, ensuring the preservation of the structural integrity of bioactive compounds.

An additional benefit of UAE is its non-thermal nature, making it suitable for the extraction of thermally sensitive compounds, effectively preventing potential degradation during the process. This aspect further contributes to the retention of the beneficial properties of polyphenols [29,30]. Given these promising attributes, the use of UAE holds considerable potential to augment the nutraceutical and functional characteristics of food products sustainably.

This research aims to establish a UAE-based method for obtaining extracts enriched in anthocyanins, showcasing significant antioxidant activities from wine lees. By achieving this objective, the study endeavors to transform this troublesome waste material into a highly valuable resource with versatile applications across various sectors, including nutraceuticals, medicine, cosmetics, and the agri-food industry. The successful implementation of this method aligns with the principles of the circular economy, as it effectively repurposes a substantial volume of waste that would otherwise be discarded annually without clear utilization prospects. Moreover, it addresses the environmental concerns associated with the treatment and management of such waste, thereby mitigating potential pollution impacts, especially by using an environmentally friendly technique such as UAE.

## 2. Materials and Methods

### 2.1. Samples

The wine lees utilized for the method optimization belonged to the Syrah variety (*Vitis vinifera*) and were procured after undergoing malolactic fermentation (designated as Sy_Ly). These lees were provided by the Forlong Winery located in Jerez de la Frontera, Spain. The removal of water from the lees was accomplished using a VirTis BenchTop Pro Freeze Dryer (SP Industries, Warminster, PA, USA), followed by a grinding of the dried lees with an electric MKM6003 coffee grinder (BSH Electrodomésticos España S.A., Zaragoza, Spain). The resulting powder was stored at −20 °C until subjected to analysis.

After optimizing the method, its validation was performed by its application to real samples. This involved applying the developed method to the sample utilized during the optimization process, as well as to another sample from the Syrah variety after malolactic fermentation subjected to drying at 40 °C (designated as Sy_Dr), instead of lyophilization. This particular sample was also obtained from the Forlong Winery. Furthermore, the method was applied to four additional wine lees samples from the Cabernet Sauvignon variety (*Vitis vinifera*) and provided by the Federal University of Bahia (Salvador, Brazil). Among these samples, two were acquired after malolactic fermentation (one subjected to lyophilization and the other to dry at 40 °C), while the other two were obtained after alcoholic fermentation (again, one lyophilized and the other dried at 40 °C). Detailed information regarding these samples and their corresponding codes can be found in Table 1.

### 2.2. Chemicals and Reagents

The solvents utilized in the extraction process consisted of Milli Q water, acquired from a Millipore water purification system (Bedford, MA, USA), and absolute ethanol (EtOH) of HPLC purity (Scharlau, Sentmenat, Spain). To adjust the pH levels of the extraction solvents, 1 M HCl and 0.5 M NaOH solutions from Panreac (Barcelona, Spain) were employed.

For the chromatographic separation and subsequent quantification of polyphenols and anthocyanins, acetonitrile and formic acid (Panreac, Barcelona, Spain), acetic acid (Merck KGaA, Darmstadt, Germany), and methanol (MeOH) (Fisher Scientific, Loughborough, UK), all at a HPLC-grade level, were used. The reference standards employed for the quantification of polyphenols and anthocyanins were quercetin 3-*O*-glucoside and cyanidin chloride, respectively, both with a purity of 95% from Sigma-Aldrich Chemical Co. (St. Louis, MO, USA). In order to determine the antioxidant activity of the extracted compounds, 6–hydroxyl-2,5,7,8-tetramethylchroman-2-carboxylic acid (Trolox) from Sigma-Aldrich (Steinheim, Germany) was employed as a standard, and 2,2-diphenyl-1-picrylhydrazyl (DPPH) (Sigma-Aldrich, St. Louis, MO, USA) was used as the reagent to evaluate radical scavenging.

### 2.3. Ultrasound-Assisted Extraction (UAE)

#### 2.3.1. Ultrasound System

The extraction process from wine lees was conducted using a UP200S ultrasonic device manufactured by Hielscher Ultrasonics GmbH (Teltow, Germany), which was coupled to a water bath (J. P. Selecta, Abrera, Spain). 

The procedure involved weighing the appropriate amount of the sample and then combining it with 25 mL of solvent, with the EtOH percentage and pH adjusted as needed. Subsequently, the extraction vessel was equipped with a probe, ensuring it did not come into contact with the flask walls. Ultrasound waves were then applied for 15 min, using the specified amplitude and cycle values. The selection of solvent volume and extraction time was based on previous experiments conducted on comparable matrices and compounds [27,29,31]. After the completion of the extraction process, the resulting mixture was subjected to centrifugation at 6810× *g* for 5 min and at a temperature of 4 °C, utilizing a centrifuge manufactured by J. P. Selecta (Abrera, Spain). The supernatant obtained from centrifugation was separated and then adjusted to a final volume of 25 mL to standardize the volume for all extractions. Before analysis, the resulting mixture was filtered through nylon syringe filters with a pore size of 0.22 µm to ensure proper filtration.

#### 2.3.2. Optimization Procedure

The investigation into the effect of the UAE variables was conducted using a Box–Behnken Design–Response Surface Methodology (BBD–RSM). This specific methodology employs a quadratic design with a three-level incomplete factorial approach. Each independent variable is set at three levels: −1 (lowest level), 0 (intermediate level), and 1 (highest level). This design generates extensive data sets suitable for analysis [32], with the exclusion of axial points to achieve a spherically distributed design. As a result, fewer experiments are required compared to commonly used orthogonal designs [33], as extreme experimental values are omitted. This advantage ensures the maintenance of mild conditions, thereby preventing any degradation of thermolabile bioactive compounds or excessive power demands.

For this study, six independent variables associated with UAE were carefully chosen for evaluation and optimization: %EtOH (25-50-75%), extraction temperature (10-35-60 °C), amplitude (30-50-70% of the maximum amplitude (70 W)), cycle (0.2-0.6-1.0 s^–1^), pH (2-5-8), and ratio (0.25-0.50-0.75 g/20 mL solvent). The choice of variables and their respective ranges were based on the group’s prior experience and in the available literature about wine lees [19,34,35]. The BBD–RSM involved a total of 54 experiments (as shown in Table 2) that were carried out randomly.

Two response variables were chosen to assess the impact of the extraction-related variables and optimize the conditions to maximize their respective concentrations: total anthocyanins concentration and antioxidant activity. It is important to emphasize that this research aims to obtain extracts enriched in bioactive compounds, whose health-related properties have been widely described, but which, in this study, have also been measured by means of antioxidant activity, in order to ensure their possible application in multiple fields. The methodologies employed to obtain the quantification of these response variables will be described below.

A mathematical model that fits a second-order polynomial function (Equation (1)) was generated for each response variable and data, resulting from the analysis:

(1) $$\begin{array}{l} \;\;Y=\beta_{0}+\beta_{1}X_{1}+\beta_{2}X_{2}+\beta_{3}X_{3}+\beta_{4}X_{4}+\beta_{5}X_{5}+\beta_{6}X_{6}+\beta_{12}X_{1}X_{2}+\beta_{13}X_{1}X_{3}+\\ \beta_{14}X_{1}X_{4}+\beta_{15}X_{1}X_{5}+\beta_{16}X_{1}X_{6}+\beta_{23}X_{2}X_{3}+\beta_{24}X_{2}X_{4}+\beta_{25}X_{2}X_{5}+\beta_{26}X_{2}X_{6}+\beta_{34}X_{3}X_{4}+\\ \beta_{35}X_{3}X_{5}+\beta_{36}X_{3}X_{6}+\beta_{45}X_{4}X_{5}+\beta_{46}X_{4}X_{6}+\beta_{56}X_{5}X_{6}+\beta_{11}{X_{1}}^{2}+\beta_{22}{X_{2}}^{2}+\beta_{33}{X_{3}}^{2}+\beta_{44}{X_{1}}^{4}+\\ \beta_{55}{X_{5}}^{2}+\beta_{66}{X_{6}}^{2} \end{array}$$

where Y is the corresponding response (total anthocyanins concentration or antioxidant activity), and β_0_ corresponds to the ordinate, whereas X_1_ (% EtOH in the solvent), X_2_ (extraction temperature), X_3_ (amplitude), X_4_ (cycle), X_5_ (pH), and X_6_ (ratio) are independent variables. Lastly, β_i_ corresponds to the linear coefficients, β_ij_ to the cross-product coefficients, and β_ii_ to the quadratic coefficients. To determine the influence of each selected independent variable and its interaction with the response variables, a second-order mathematical model was established. Additionally, surface graphs were generated to visualize the relationship between the variables and the response. The optimal levels of the influential variables were identified using the software application Statgraphic Centurion (version XVII) from Statgraphics Technologies, Inc. (The Plains, VA, USA). Moreover, an analysis of variance (ANOVA) was conducted to assess the significance of the effects of the variables and their interactions with the response variables. The application of this software facilitated the comprehensive analysis and interpretation of the experimental data and allowed for the determination of optimal extraction conditions for maximum efficiency.

### 2.4. Total Anthocyanins Concentration

#### 2.4.1. Identification of Anthocyanins by UHPLC-PDA-QToF-MS

For the identification of anthocyanins in wine lees, an ultra-high-performance liquid chromatography equipment coupled to a photodiode array detector and a quadrupole-time-of-flight mass Spectrometer (UHPLC–PDA-QToF–MS) model Xevo G2 from Waters Corp. (Milford, MA, USA) was employed. The UHPLC system consisted of a 100 × 2.1 mm reverse-phase C18 analytical column (Acquity UPLC BEH C18, Waters) with a particle size of 1.7 µm. The mobile phase A contained a mixture of water and formic acid at a concentration of 2%, while phase B consisted of pure MeOH. The flow rate during the analysis was set at 0.4 mL/min.

The following gradient was applied for injection (%B): 5%, 0 min; 20%, 3.30 min; 30%, 3.86 min; 40%, 5.05 min; 55%, 5.35 min; 60%, 5.64 min; 95%, 5.94 min; and 95%, 7.50 min. Each analysis required a total time of 12 min, including 4 min for re-equilibration. 

Electrospray ionization was employed in positive ionization mode. The desolvation gas temperature was set at 500 °C with a flow rate of 700 L/h, and the capillary cone was set at 700 V. The cone gas flow was 10 L/h, the source temperature was maintained at 150 °C, and a cone voltage of 20 V was applied. Additionally, the trap collision energy was set at 4 eV.

For anthocyanin identification, a full-scan mode was utilized in a mass range of 100–1200 *m*/*z*. This comprehensive analytical approach allowed for the accurate and efficient detection of the anthocyanins present in wine lees samples. A total of 11 anthocyanins were identified in the wine lees extracts [M^+^]: Petunidin 3-*O*-glucoside (*m*/*z* 479.1190), peonidin 3-*O*-glucoside (*m*/*z* 498.1288), malvidin 3-*O*-glucoside (*m*/*z 528.1035*), cyanidin 3-(6″-acetylglucoside) (*m*/*z* 491.1190), petunidin 3-(6″-acetylglucoside) (*m*/*z* 521.4295), peonidin 3-(6″-acetylglucoside) (*m*/*z* 505.1346), malvidin 3-(6″-acetylglucoside) (*m*/*z* 535.5465), malvidin 3-(6″-*p*-caffeyglucoside) (*m*/*z* 655.6539), cyanidin 3-*O*-(6″-*O*-*p*-coumaroyl-glucoside) (*m*/*z* 595.5165), petunidin 3-(6″-*p*-coumaroyl-glucoside) (*m*/*z* 787.2054), and peonidin 3-(6″-*p*-coumaroyl-glucoside) (*m*/*z* 769.2347). The identified anthocyanins are in agreement with those described by Duarte et al. [18] and Romero-Díez et al. [36] in wine lees samples.

#### 2.4.2. Separations and Quantification of Anthocyanins by UHPLC–UV–vis

The separation and quantification of the identified anthocyanins in wine-lees samples were performed using an Elite UHPLC LaChrom System from Hitachi (Tokyo, Japan). This system included an L-2200U autosampler, an L2300 column oven set at 50 °C, two L-2160U pumps, and a UV–Vis detector L-2420U, which was set to 520 nm for identification purposes. A reversed-phase C18 column (Phenomenex, Kinetex, CoreShell Technology, Torrance, CA, USA) with dimensions of 2.1 × 50 mm and 2.6 µm particle size was utilized. The mobile phase A consisted of water with 5% formic acid, while phase B was pure MeOH, both filtered through a 0.22 µm filter (RephiLe Bioscience, Ltd., Shanghai, China) and degassed using an ultrasonic bath (Elma S300, Elmasonic, Singen, Germany) before use. The flow rate was set at 0.7 mL/min.

Prior to analysis, the extracts underwent filtration through a 0.22 µm nylon syringe filter (Membrane Solutions, Dallas, TX, USA). The injection volume for UHPLC separation was set at 15 μL, and a gradient was applied for separation (%B): 2% at 0.00 min; 15% at 3.30 min; 35% at 4.80 min; 100% at 6.00 min.

The selection of this UHPLC method was due to its efficiency in separating the eleven major anthocyanins identified in the samples in less than 7 min. This time-saving feature is particularly beneficial for quality control laboratories that require a large number of analyses to be performed daily.

For quantification, cyanidin chloride was chosen as the reference standard, and a calibration curve (y = 300568.88x − 28462.43) with a coefficient of regression (R^2^) of 0.9999 was constructed. The limits of detection (LOD) and quantification (LOQ) were determined to be 0.198 mg L^−1^ and 0.662 mg L^−1^, respectively. The normal distribution of residuals was assessed using the Shapiro–Wilk test, yielding a W value of 0.8514 (very close to 1) and a *p*-value of 0.803 (above 0.05), confirming the hypothesis H0. The assumption of similar absorbance levels for different anthocyanins, considering their individual molecular weights, allowed for the use of this calibration curve to prepare a calibration curve for each identified anthocyanin. To ensure accuracy and reproducibility, all analyses were conducted in duplicate. A list of retention times for each anthocyanin and its corresponding calibration curves can be found in Table 3.

The determination of total anthocyanins was achieved by calculating the sum of the quantities of the eleven identified anthocyanins. This cumulative value of total anthocyanins was then expressed in mg per 100 g of a dry-weighted sample. This cumulative value of total anthocyanins served as the response variable during the optimization process.

### 2.5. Antioxidant Activity

As was previously mentioned, the antioxidant activity of the wine lees extracts has also been considered the response variable. Antioxidant activity was assessed using DPPH assays, following the method described by Brand-Williams et al. [37], with some modifications as suggested by Miliauskas et al. [38]. Trolox was employed as the standard, and a six-point linear regression model was constructed ranging from 0 to 1.4 mM, with triplicate measurements at each concentration. The obtained regression equation (y = 88.94x + 0.75) exhibited a high regression coefficient of R^2^ = 0.9959. The antioxidant activity was expressed as mg of trolox equivalents (TE) per gram of the dry-weighted sample (mg TE/g dw). This measurement provides an indication of the antioxidant capacity of the wine lees extracts, which is important for assessing their potential health benefits and functional properties—objectives of this research.

### 2.6. Repeatability and Intermediate Precision Study

The optimized method’s suitability and accuracy were assessed through repeatability and intermediate precision tests. For the repeatability evaluation, nine experiments were conducted on the same day, aiming to determine the method’s precision when applied multiple times within a short time frame. On the other hand, to determine the intermediate precision of the method, nine additional extractions were performed on two consecutive days, totaling 27 extractions, all under the optimized conditions. 

The statistical parameters selected to evaluate the suitability and accuracy of the developed method were the coefficients of variation (CV). 

### 2.7. Data Analysis

The BBD–RSM design was conducted using the Statgraphic Centurion software (version XVII) from Statgraphics Technologies, Inc. (The Plains, VA, USA). The data obtained were compared and grouped based on the least significant difference (LSD) method, response surface regression techniques, analysis of variance (ANOVA) and the Fisher test. The significance level was set at 95%, corresponding to a *p*-value ≤ 0.05.

RStudio software version 4.2.2 (RStudio Team 2022, Boston, MA, USA) was employed for non-supervised Hierarchical Cluster Analysis (HCA) using the stats package.

## 3. Results and Discussion

### 3.1. BBD–RSM Method Optimization 

In this study, the influence of various UAE variables and their optimization for total anthocyanins concentrations and the antioxidant activity of the extracts were investigated using BBD–RSM. Six extraction-related variables were carefully selected for evaluation, including the %EtOH, temperature, amplitude, cycle, pH, and ratio. The experimental design comprised 54 randomly performed extraction experiments based on the chosen variables. Subsequently, the obtained extracts were treated and analyzed for the two selected response variables (total anthocyanins concentrations and antioxidant activity of the extracts).

#### 3.1.1. Total Anthocyanin Concentration

The determination of total anthocyanin concentration obtained from UAE extraction was selected as a response variable. To ascertain the concentration of individual anthocyanins, an HPLC analysis was conducted on the extracts (Section 2.4), and the values were aggregated to compute the overall concentration of total anthocyanin content (mg/100 g dw) for each of the 54 experiments.

The BBD–RSM methodology was applied to analyze this response variable, and its correlation with the predicted values revealed an average deviation of 1.55%, ranging from 0.05% to 5.02%. Moreover, the developed model demonstrated an R^2^ value of 0.99. The Durbin–Watson *p*-value in the ANOVA table (2.40) exceeded 0.05, indicating an absence of significant disparities between the predicted and observed values. Consequently, the model was deemed adequate for predicting the total anthocyanin concentration in wine lees extracts.

**Figure 1:**
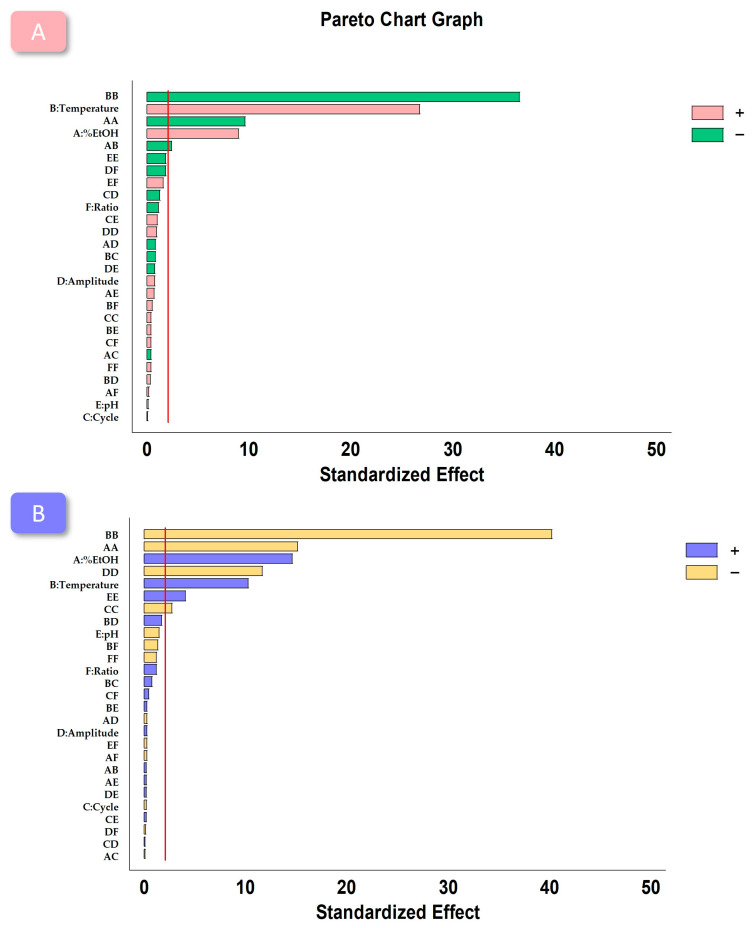
Pareto charts obtained from BBD–RSM analysis for (**A**) total anthocyanin concentration; and (**B**) the antioxidant activity of the obtained extracts by UAE. Vertical red line indicates the 95% of confidence.

In order to identify influential variables affecting the extraction of total anthocyanins from wine lees, a *t*-test with a confidence level of 95% was conducted. The results (Table 4) revealed that the following variables significantly influenced the total anthocyanin extraction: %EtOH in the solvent (*p*-value: 0.000), extraction temperature (*p*-value: 0.000), quadratic interaction of %EtOH in solvent (*p*-value: 0.000), quadratic interaction of extraction temperature (*p*-value: 0.000), and interaction of %EtOH and extraction temperature (*p*-value: 0.0252). These results were visually depicted in a pareto chart for enhanced comprehension (Figure 1A). It is worth noting that the %EtOH and extraction temperature variables exhibited a positive effect on total anthocyanin extraction, i.e., higher values within the studied ranges led to increased total anthocyanin content in the analyzed extracts.

A second-order polynomial equation (Equation (2)) was employed to determine the total anthocyanin concentration (Y) under optimal conditions, established based on the coefficients obtained through the BBD–RSM analysis:

(2) $$\begin{array}{l} \;\;Y=-12.373+1.911\cdot X_{1}+4.882\cdot X_{2}+4.844\cdot X_{3}+0.207\cdot X_{4}+0.376\cdot X_{5}-\\ 6.023\cdot X_{6}-0.015\cdot {X_{1}}^{2}-0.004\cdot X_{1}\cdot X_{2}-0.04\cdot X_{1}\cdot X_{3}-0.001\cdot X_{1}\cdot X_{4}+0.010\cdot X_{1}\cdot X_{5}+\\ 0.032\cdot X_{1}\cdot X_{6}-0.057\cdot {X_{2}}^{2}-0.093\cdot X_{2}\cdot X_{3}+0.001\cdot X_{2}\cdot X_{4}+0.004\cdot X_{2}\cdot X_{5}+0.082\cdot X_{2}\cdot X_{6}+\\ 2.376\cdot {X_{3}}^{2}-0.171\cdot X_{3}\cdot X_{4}+0.937\cdot X_{3}\cdot X_{5}+2.812\cdot X_{3}\cdot X_{6}+0.002\cdot {X_{4}}^{2}-0.014\cdot X_{4}\cdot X_{5}-\\ 0.401\cdot X_{4}\cdot X_{6}-0.199\cdot {X_{5}}^{2}+2.353\cdot X_{5}\cdot X_{6}+5.282\cdot {X_{6}}^{2} \end{array}$$

The optimal conditions for the maximum recovery of total anthocyanin concentration from wine lees were 0.20 g of a sample extracted with 25 mL of solvent at 60% EtOH in water with a pH of 4.40 at 60 °C and using an amplitude and cycle of 70% and 0.2 s^–1^, respectively. The percentage of EtOH in the solvent aligns with the approaches employed by other researchers in the extraction of anthocyanins from wine lees. For instance, Costa-Pérez et al. [10] employed an acidified mixture of 50% MeOH in water for their extraction process. Similarly, Tagkouli et al. [23] also used a 50% mixture of EtOH in water for their extraction, albeit at a higher extraction temperature of 85 °C, which would require higher energetic consumption.

#### 3.1.2. Antioxidant Activity

As previously mentioned, one of the primary objectives of this research is to obtain enriched extracts from wine lees with potential applications in various fields, such as nutraceuticals, medicine, or the pharmaceutical industry. Hence, the presence of desirable properties, such as antioxidant activity, is of great interest. To evaluate the antioxidant activity of the 54 obtained extracts from the experimental design, the DPPH methodology was utilized, and the measured antioxidant activity was employed as a response variable. This antioxidant activity was then correlated with the predicted values obtained from the model (Table 1). The analysis revealed an average difference of 2.33% with variations ranging from 0.01% to 11.59%. The developed method exhibited an R^2^ value of 0.98, and the Durbin–Watson *p*-value (2.82) in the ANOVA table was found to be greater than 0.05. This indicated that there were no significant discrepancies between the predicted and observed values, confirming the model’s adequacy for predicting the antioxidant activity of the wine lees extracts.

A *t*-test was performed at a confidence level of 95% to assess the influence of the selected variables on the antioxidant activity of the extracts. Among the variables studied, %EtOH in the solvent (*p*-value: 0.000), extraction temperature (*p*-value: 0.000), the quadratic interaction of %EtOH in solvent (*p*-value: 0.000), the quadratic interaction of extraction temperature (*p*-value: 0.000), the quadratic interaction of amplitude (*p*-value: 0.000), the quadratic interaction of cycle (*p*-value: 0.011), and the quadratic interaction of pH (*p*-value: 0.004) were found to be influential in the antioxidant activity of the extracts, as shown in Table 4. The results were further illustrated using a pareto chart graph (Figure 1B), where it can be observed that the influence of the %EtOH and temperature were positive, as in the case of the anthocyanins. To determine the antioxidant activity (Y) under optimal conditions, as established based on the coefficients obtained through the BBD–RSM analysis, the second-order polynomial equation (Equation (3)) was utilized. This equation is useful for predicting the antioxidant activity of the wine lees extracts under the identified optimal conditions, facilitating their application in various industries and fields where antioxidant properties are highly valuable:

(3) $$\begin{array}{l} \;\;Y=-8.965+0.201\cdot X_{1}+0.332\cdot X_{2}+1.038\cdot X_{3}+0.200\cdot X_{4}-0.340\cdot X_{5}+\\ 2.187\cdot X_{6}-0.002\cdot {X_{1}}^{2}+2.4\cdot 10^{-5}\cdot X_{1}\cdot X_{2}-0.001\cdot X_{1}\cdot X_{3}-2.8\cdot 10^{-5}\cdot X_{1}\cdot X_{4}-2.7\cdot 10^{-4}\cdot X_{1}\cdot X_{5}\\ +0.002\cdot X_{1}\cdot X_{6}-0.005\cdot {X_{2}}^{2}+0.006\cdot X_{2}\cdot X_{3}+2.7\cdot 10^{-4}\cdot X_{2}\cdot X_{4}+1.9\cdot 10^{-4}\cdot X_{2}\cdot X_{5}-\\ 0.017\cdot X_{2}\cdot X_{6}-1.208\cdot {X_{3}}^{2}+7.8\cdot 10^{-4}\cdot X_{3}\cdot X_{4}+0.010\cdot X_{3}\cdot X_{5}+0.256\cdot X_{3}\cdot X_{6}-0.002\cdot {X_{4}}^{2}+\\ 2.3\cdot 10^{-4}\cdot X_{4}\cdot X_{5}-0.002\cdot X_{4}\cdot X_{6}+0.032\cdot {X_{5}}^{2}-0.027\cdot X_{5}\cdot X_{6}-1.393\cdot {X_{6}}^{2} \end{array}$$

The optimal conditions to achieve the maximum antioxidant activity from wine lees were 0.57 g of the sample in 25 mL of solvent with 60% of EtOH at pH 2, extracted at 37 °C with an amplitude of 50% and 0.6 s^–1^ of the cycle. The exhaustive comparison of extraction conditions with existing literature was a challenge because the main objective of most studies was the extraction of bioactive compounds, with its associated properties being a supplementary consideration and not the main goal of optimization. Nevertheless, the notable decrease in the optimal pH value was a significant finding for the researchers. This observation could be highly related to the pH dependence of antioxidant activity. Numerous authors have stated that antioxidant activity is influenced by pH through several mechanisms: (1) electrochemical oxidation and the involvement of H^+^ ions; (2) correlation between the torsion angle of one ring with the rest of the molecules and scavenging activity due to increased conjugation afforded by planarity; (3) the oxidation stability of the compound; and (4) the transformation of the compound [39,40]. 

As observed, certain similarities were found among the influential variables affecting the total anthocyanins concentration and antioxidant activities in the analyzed extracts. However, significant dissimilarities, especially in the optimal values of these variables, were also identified. Therefore, a multi-response model was evaluated to develop a single model that could maximize the two response variables simultaneously. The desirability obtained was significantly higher than expected, proving the possibility of using a unique model that allows for obtaining the highest anthocyanin content with antioxidant activity from wine lees. The optimal conditions of this method were 0.25 g of the sample extracted with 25 mL of solvent (50% EtOH and pH 2) at 40 °C using an amplitude of 53% and a cycle of 0.30 s^–1^.

### 3.2. Optimal Extraction Time of the Method

Finally, a kinetic study was carried out in order to evaluate the optimal extraction time of the combined model developed and to assess the influence of the extraction times on the concentration of total anthocyanins obtained and the antioxidant activity of the extracts.

For this purpose, the following extraction times were considered: 2, 5, 10, 15, 20, and 30 min. The extractions were performed in triplicate, following the optimal conditions of the combined method. After the extraction process, the extracts were treated as previously described and analyzed using the established methodologies to quantify the concentrations of total anthocyanins, as well as to measure the antioxidant activity. 

**Figure 2:**
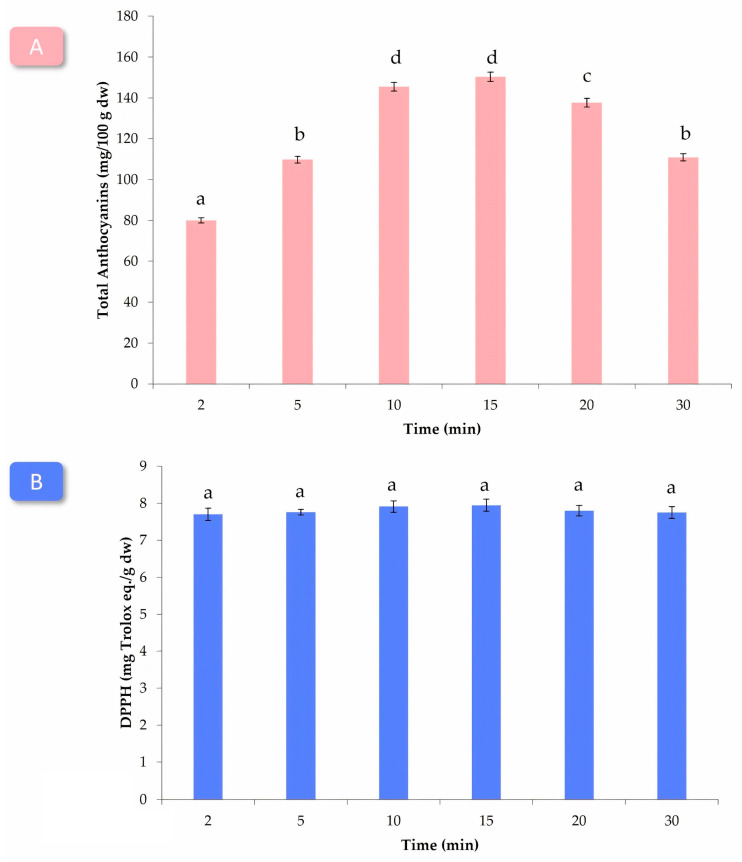
(**A**) Total anthocyanin concentrations (mg/100 g dw) and (**B**) antioxidant activity (mg Trolox eq./g dw) of the extracts according to the different extraction times (*n* = 3). Different letters indicate significant differences at a 95% confidence.

The average total anthocyanin concentration was calculated for each extraction time and represented in Figure 2A. The concentrations of anthocyanins increased steadily, reaching their maximum at the 15 min mark. However, beyond this point, the concentration started to decrease, likely due to the thermodegradation of the anthocyanin compounds [41]. Statistical analysis was conducted using ANOVA with a confidence level of 95%, resulting in a *p*-value below 0.05, which confirmed the significant influence of the extraction time on anthocyanin concentrations. Although there were no statistically significant differences between the 10 min and 15 min extraction times, the 10 min duration was selected as the optimal extraction for anthocyanin recovery. This choice was likely based on practical considerations and low energy consumption.

Interestingly, the extraction time did not appear to be a significant factor influencing the antioxidant activity of the extracts. This observation was further confirmed through ANOVA analysis at a 95% confidence level, revealing no statistically significant differences between the various extraction times. Consequently, a 10 min extraction time was selected as the optimal duration for maximizing the anthocyanins concentration extracted and the antioxidant activity of the wine lees extracts. This abbreviated extraction time is advantageous for practical applications, as it allows for a rapid and efficient extraction process to obtain significant antioxidant activity levels.

Indeed, the chosen short extraction time offers significant advantages compared to the typically employed durations in studies focused on extracting bioactive compounds from wine lees. For instance, Costa-Pérez et al. [10], Tapia Quirós et al. [42], or Dujmic et al. [34] utilized extraction times of 60, 30, and 25 min, respectively, for the extraction of polyphenols from wine lees using UAE. By contrast, the 2 min extraction time selected in this study represents a considerable reduction in the overall extraction process while still achieving comparable results. Moreover, this short extraction time aligns with the approach adopted by De Luca et al. [19] who achieved the successful extraction of polyphenols in just 15 min. In conclusion, the developed methodology provides a rapid and reliable means of obtaining anthocyanin-rich extracts from wine lees while maintaining their beneficial bioactive properties, including antioxidant activity.

### 3.3. Optimal Conditions

At this stage, one extraction method was optimized to obtain the highest anthocyanin concentrations with significant antioxidant activity from wine lees extraction using USE. In order to evaluate this methodology, six extractions were performed using 0.25 g of the sample extracted with 25 mL of the solvent (50% EtOH and pH 2) at 40 °C, using an amplitude of 53% and a cycle of 0.30 s^–1^ for 10 min. 

The extracts obtained were analyzed, and the eleven anthocyanins previously identified were quantified; the sum of the concentrations was calculated, obtaining the total anthocyanin concentration. This resulted in extracts with a total anthocyanin concentration of 148.03 ± 1.71 mg/100 g dw. 

Comparison with existing literature reveals that the developed method yielded significantly higher anthocyanin concentrations compared to other reported extraction methods. For example, Costa-Pérez et al. [10] evaluated the anthocyanin concentration in var. Monastrell wine lees, using for this, 100 mg of a sample that was mixed with 1 mL of an acidified mixture of 50% MeOH in water, sonicated for 60 min, kept overnight, and sonicated again for 60 min. In this case, the anthocyanin concentration achieved was 32.70 mg/100 g of the sample. As can be observed, the concentrations obtained were significantly lower than those achieved with the developed method and under softer conditions. Sancho-Galán et al. [43] also optimized a method for the extraction of anthocyanins from var. Tempranillo using UAE. The conditions obtained were in agreement with many of those obtained in the developed method; 0.5 g of the sample were extracted with 11 mL of solvent (49% MeOH, pH 6.9) at 10 °C and using an amplitude and cycle of 51% and 0.7 s^–1^, respectively. While the percentage of alcoholic solvent and the amplitude used were similar to the developed method, the required temperature was significantly lower. However, the total anthocyanin concentration achieved in their study remained in the range of 90 mg/L, lower than that obtained in this research. In conclusion, the developed extraction method demonstrated superior efficiency in achieving higher anthocyanin concentrations from wine lees compared to previously reported methods in the literature. This was accomplished under milder extraction conditions, making it a promising approach for extracting anthocyanins from wine lees.

On the other hand, the antioxidant activity of the extracts obtained was also evaluated using DPPH methodology. The average activity obtained was 7.87 ± 0.32 mg TE/g dw.

In comparison with existing literature, it is challenging to find direct matches for antioxidant activity optimization research, as most studies focus on the extraction of bioactive compounds, with antioxidant properties being a consequence rather than the main objective. However, some studies have evaluated the antioxidant activity of extracts from wine lees. For instance, Romero-Díez et al. [36] evaluated the antioxidant activity of the extracts obtained from different *Vitis vinifera* varieties of wine lees from Portugal, achieving 4952 µmol/g sample as an antioxidant activity. Sato et al. [44] explored the antioxidant activity of the wine lees used as feed for ruminates. For that, 0.5 g of wine lees was incubated with 40 mL of a buffer solution at 39 °C for 48 h, obtaining an antioxidant activity of the extracts of 13.2 µmol/g sample. Lastly, Ciliberti et al. [20] also evaluated the use of wine less as an immune modulator. For that, wine lees samples were extracted with a MAE methodology, which required an ethanol solution (50%) at different temperatures (50–200 °C), for 15 min. The extracts exhibited antioxidant activities that ranged from 12.36 to 467.4 µmol/g sample. Overall, despite the challenges in finding direct comparisons, the antioxidant activities observed in the extracts from the second optimized method are consistent with those reported in the literature for similar studies on wine lees.

### 3.4. Repeatability and Intermediate Precision

In the subsequent phase, the repeatability and intermediate precision of the optimized method were evaluated to ensure the accuracy and precision of the developed technique for obtaining anthocyanin-enriched extracts from wine lees with significant antioxidant activities. Repeatability was assessed by conducting nine extractions on the same day, under the established optimal conditions. Similarly, for the evaluation of intermediate precision, nine extractions were performed on each of two consecutive days, resulting in a total of 27 extractions (*n* = 9 + 9 + 9).

The total concentration of anthocyanins in the extracts was determined using the previously established procedures, and the average total anthocyanin concentration (mg/100 g dw), along with its corresponding standard deviation, were calculated. Additionally, the antioxidant activity of the extracts was measured, and the average activity (mg Trolox equivalents/g dw) and standard deviation were computed accordingly. The specific numerical values obtained from the repeatability and intermediate precision evaluations can be found in Table 5.

Regarding the total anthocyanins, the relative standard deviation (RSD) resulting from the repeatability and intermediate precision tests were found to be 0.73% and 0.92%, respectively. Similarly, for the antioxidant activity, the RSD values obtained were 2.35% and 3.29% for the repeatability and intermediate precision tests, respectively. It is important to note that all RSD values obtained were below 5%. This outcome confirms the high precision level of the optimized methods employed in this study. The low RSD values indicate that the developed technique is reliable and can consistently produce anthocyanins-enriched extracts from wine with significant antioxidant activities. These findings demonstrate the robustness and accuracy of the method, making it suitable for practical applications in the extraction and quantification of anthocyanins from wine lees.

### 3.5. Application of the Optimized Method to Real Samples

Once the precision properties of the developed methods were confirmed, the next step was the application of the developed method to real samples of wine lees. Samples from Syrah and Cabernet Sauvignon varieties were used for the analysis (Table 1) under optimal conditions. The total anthocyanins concentrations were measured using the UHPLC-PDA system, and the antioxidant activities were measured using DPPH methodology.

Significant differences were found in the concentrations of bioactive compounds. The average total anthocyanin concentrations ranged from 27.72 to 148.03 mg/100 g, with the highest concentration observed in the Syrah lees with lyophilization treatment (sample used for method optimization) and the lowest concentration observed in the Cabernet Sauvignon sample after alcoholic fermentation and drying at 40 °C (Table 6). These concentrations are consistent with literature reports for Syrah and Cabernet Sauvignon wine lees, which have anthocyanin concentrations in the range of 36.4–390.67 and 0.10–587.00 mg/100 g, respectively [16,45,46,47,48].

A multifactorial ANOVA was conducted with the total anthocyanin concentration data. The analysis revealed that the variety significantly influenced the total anthocyanin concentrations (*p*-value: 6.791·10^–7^). Additionally, the type of pretreatment before extraction (lyophilization or drying at 40 °C) also had a significant effect on anthocyanin concentration (*p*-value: 3.000·10^–6^). Furthermore, the fermentation undergone by the samples was influential for the total anthocyanin amount(*p*-value: 0.005), which was expected, as previous studies have shown that a second fermentation (malolactic fermentation) enhances the accumulation of bioactive compounds [49]. The interaction between the three factors (variety, fermentation, and treatment) was also found to be influential in the total anthocyanin concentration in the final extracts (*p*-value: 0.010)

The antioxidant activity of the real samples was also evaluated, with the highest antioxidant activity observed in the Syrah wine lees employed for the method development (7.82 mg/100 g dw) and the lowest activity in the Cabernet Sauvignon sample after alcoholic fermentation (4.60 mg/100 g dw) (Table 6). These antioxidant activities are consistent with those reported in the literature for Syrah and Cabernet Sauvignon varieties, which exhibited ranges of 0.384–4.69 and 1.09–3.93 mmol TE/g dw, respectively [11,16,45,48].

A multifactorial ANOVA was again conducted, and the variety (*p*-value: 0.006), treatment (*p*-value: 6.254·10^–5^), and fermentation (*p*-value: 2.498·10^–4^) were found to be significantly influential variables in the antioxidant activity of the extracts. Additionally, the interaction between these three factors (*p*-value: 0.004) was also determined to have a significant impact on the antioxidant activity.

These findings demonstrate that the variety of grapes used, the treatment method (lyophilization or drying at 40 °C), and the fermentation process all play significant roles in determining the antioxidant activity of the extracted compounds from wine lees. The results also highlight the suitability and effectiveness of the developed method in quantifying anthocyanins and assessing antioxidant activity in wine lees extracts, making it a valuable tool for studying the bioactive properties of these by-products in the winemaking industry.

**Figure 3:**
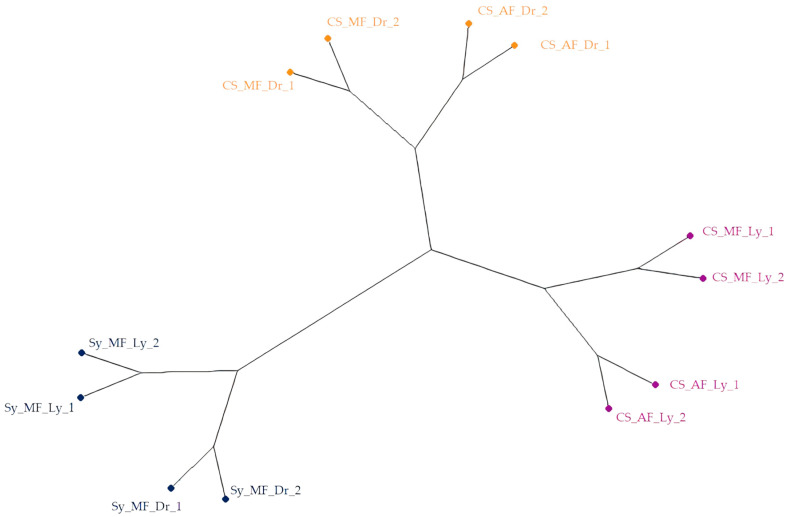
Dendrogram obtained from the HCA of Syrah and Cabernet Sauvignon wine lees samples (D_12×2_). The samples are colored according to the three principal clusters.

Finally, a Hierarchical Cluster Analysis (HCA) was conducted with the samples to observe their distribution using a non-supervised statistical technique. Ward’s method with squared-Euclidean distance was applied, and the dendrogram was represented in a phylogenetic tree (Figure 3). As observed, the major distance between the samples was established based on the variety, with the Syrah variety samples forming a distinct group separate from the Cabernet Sauvignon samples. Next, the second major distance was established based on the treatment method, with the lyophilized samples completely separated from the dried samples. Finally, the fermentation process appeared to be the least determining factor in the distribution of the samples, although it did cause some grouping of the samples according to their nature.

In conclusion, the influence of the three factors (variety, treatment, and fermentation) on the bioactive compounds and health-related properties of the extracts was evident. The Syrah variety, malolactic fermentation, and lyophilization treatment were found to be the best conditions for obtaining the highest concentrations of anthocyanins and antioxidant activities in the extracts. The results from the HCA supported the findings from the ANOVA analysis, further validating the significance of these factors in determining the bioactive properties of wine lees extracts.

Overall, this study demonstrated the importance of considering these factors when extracting and quantifying bioactive compounds from wine lees, and it provides valuable insights for optimizing extraction processes to obtain anthocyanin-enriched extracts with significant antioxidant activities from different grape varieties.

## 4. Conclusions

In this research, an ultrasound-assisted extraction methodology was developed to obtain bioactive compound-enriched extracts from wine lees. The influence of various USE variables on the extraction of total anthocyanins and antioxidant activity was evaluated. Significant factors affecting both anthocyanin extraction and antioxidant activity included the percentage of ethanol in the solvent, extraction temperature, and amplitude. A multiparametric method was developed achieving the highest anthocyanin concentration and antioxidant activity of the extracts under the same optimal conditions with an important desirability factor. Optimal conditions were 0.25 g of a wine lees sample extracted with 25 mL of solvent (50% EtOH and pH 2) at 40 °C using an amplitude of 53% and a cycle of 0.30 s^–1^ for 10 min. In addition, the method underwent precision and accuracy tests, demonstrating its reliability, with relative standard deviations consistently below 5%.

The developed method was also applied to real samples of wine lees from Syrah and Cabernet Sauvignon varieties with different treatments. Syrah samples generally exhibited higher concentrations of bioactive compounds and antioxidant activity compared to Cabernet Sauvignon samples. Malolactic fermentation was found to promote the accumulation of bioactive compounds and enhance their properties. Additionally, lyophilization emerged as a favorable pretreatment method for achieving both objectives. In conclusion, the research highlights the significant influence of variety, treatment, and fermentation on the concentrations and properties of bioactive compounds in wine lees extracts. 

The developed method represents an important advancement in circular economy and environmental contamination reduction, making these residues valuable resources for various applications. Overall, this study contributes to the utilization of wine lees as a source of anthocyanin-enriched extracts with significant antioxidant properties, offering potential benefits in multiple fields and promoting sustainable practices in the winemaking industry.

## Figures and Tables

**Table 1:** Wine lees samples employed in this research and their characteristics.

Variety	Origin	Fermentation	Treatment	Code
Syrah	Spain	Malolactic	Lyophilized	Sy_MF_Ly
		Malolactic	Dried at 40 °C	Sy_MF_Dr
Cabernet Sauvignon	Brazil	Malolactic	Lyophilized	CS_MF_Ly
		Malolactic	Dried at 40 °C	CS_MF_Dr
		Alcoholic	Lyophilized	CS_AF_Ly
		Alcoholic	Dried at 40 °C	CS_AF_Dr

**Table 2:** Box–Behnken design to optimize the extraction procedure of total anthocyanins and antioxidant activity from wine lees extracts using UAE.

Experiment	% EtOH	Temperature (°C)	Amplitude	Cycle	pH	Ratio	Total Anthocyanins (mg/100 g dw)			Antioxidant Activity (mg Trolox eq./g dw)		
							Observed	Adjusted	Error (%)	Observed	Adjusted	Error (%)
1	50	35	50	0.2	2	0.25	149.34	147.42	1.29	7.89	7.93	0.57
2	50	35	50	1	2	0.25	145.85	144.58	0.87	7.80	7.84	0.46
3	50	35	50	0.2	8	0.25	142.17	141.81	0.25	7.87	7.81	0.72
4	50	35	50	1	8	0.25	144.73	143.47	0.87	7.92	7.77	1.94
5	50	35	50	0.2	2	0.75	139.71	141.92	1.58	7.94	8.03	1.22
6	50	35	50	1	2	0.75	140.79	140.20	0.42	7.93	8.04	1.44
7	50	35	50	0.2	8	0.75	141.15	143.37	1.57	7.93	7.83	1.20
8	50	35	50	1	8	0.75	145.18	146.16	0.68	7.88	7.89	0.20
9	50	10	30	0.6	2	0.5	90.48	89.94	0.60	4.59	4.21	8.39
10	50	60	30	0.6	2	0.5	120.31	123.08	2.30	4.85	4.86	0.22
11	50	10	70	0.6	2	0.5	91.34	91.94	0.66	3.98	3.93	1.34
12	50	60	70	0.6	2	0.5	127.49	126.41	0.84	5.26	5.14	2.36
13	50	10	30	0.6	8	0.5	89.10	91.25	2.41	3.83	4.02	4.96
14	50	60	30	0.6	8	0.5	127.24	125.57	1.31	4.74	4.73	0.17
15	50	10	70	0.6	8	0.5	91.49	89.79	1.86	3.74	3.79	1.46
16	50	60	70	0.6	8	0.5	125.99	125.46	0.42	4.74	5.06	6.77
17	25	35	30	0.2	5	0.5	126.34	127.93	1.26	4.90	5.08	3.60
18	75	35	30	0.2	5	0.5	141.99	141.59	0.28	6.54	6.49	0.88
19	25	35	30	1	5	0.5	133.43	131.45	1.49	4.91	5.06	3.12
20	75	35	30	1	5	0.5	142.43	143.52	0.76	6.61	6.45	2.40
21	25	35	70	0.2	5	0.5	132.66	132.94	0.21	4.93	5.12	3.88
22	75	35	70	0.2	5	0.5	143.34	143.96	0.43	6.65	6.47	2.73
23	25	35	70	1	5	0.5	129.20	130.96	1.37	5.05	5.13	1.67
24	75	35	70	1	5	0.5	143.35	140.40	2.06	6.66	6.45	3.14
25	50	10	50	0.2	5	0.25	95.54	92.88	2.78	4.24	4.18	1.47
26	50	60	50	0.2	5	0.25	126.00	128.12	1.68	5.15	5.24	1.69
27	50	10	50	1	5	0.25	96.16	94.16	2.09	3.99	3.99	0.09
28	50	60	50	1	5	0.25	121.87	125.67	3.12	5.04	5.29	4.87
29	50	10	50	0.2	5	0.75	94.63	89.89	5.02	4.65	4.46	4.08
30	50	60	50	0.2	5	0.75	126.12	127.18	0.83	5.03	5.08	0.94
31	50	10	50	1	5	0.75	93.45	92.28	1.25	4.52	4.38	3.27
32	50	60	50	1	5	0.75	122.25	125.85	2.95	5.23	5.24	0.08
33	25	10	50	0.6	2	0.5	71.05	72.90	2.61	3.11	3.13	0.81
34	75	10	50	0.6	2	0.5	84.10	88.27	4.96	4.04	4.51	11.59
35	25	60	50	0.6	2	0.5	116.96	111.99	4.25	4.35	4.03	7.26
36	75	60	50	0.6	2	0.5	118.00	116.78	1.03	5.38	5.47	1.63
37	25	10	50	0.6	8	0.5	70.86	71.01	0.21	3.16	3.01	4.78
38	75	10	50	0.6	8	0.5	85.41	89.31	4.57	4.05	4.30	6.18
39	25	60	50	0.6	8	0.5	114.39	111.29	2.71	4.37	3.97	9.25
40	75	60	50	0.6	8	0.5	119.80	119.02	0.65	5.28	5.32	0.88
41	25	35	30	0.6	5	0.25	126.53	128.54	1.59	5.14	5.13	0.25
42	75	35	30	0.6	5	0.25	142.73	141.00	1.21	6.52	6.49	0.49
43	25	35	70	0.6	5	0.25	132.83	134.81	1.49	5.14	5.20	1.07
44	75	35	70	0.6	5	0.25	143.33	144.63	0.91	6.68	6.51	2.60
45	25	35	30	0.6	5	0.75	130.67	130.74	0.05	5.03	5.23	4.03
46	75	35	30	0.6	5	0.75	147.36	144.01	2.27	6.74	6.65	1.25
47	25	35	70	0.6	5	0.75	128.62	128.99	0.28	5.26	5.26	0.10
48	75	35	70	0.6	5	0.75	140.27	139.62	0.46	6.59	6.63	0.52
49	50	35	50	0.6	5	0.5	146.56	144.70	1.27	7.87	7.89	0.19
50	50	35	50	0.6	5	0.5	143.27	144.70	1.00	7.92	7.89	0.36
51	50	35	50	0.6	5	0.5	146.86	144.70	1.47	7.90	7.89	0.15
52	50	35	50	0.6	5	0.5	147.27	144.70	1.74	7.84	7.89	0.61
53	50	35	50	0.6	5	0.5	143.67	144.70	0.72	7.89	7.89	0.01
54	50	35	50	0.6	5	0.5	140.58	144.70	2.93	7.91	7.89	0.22

**Table 3:** Anthocyanins quantified by UHPLC–UV–vis in wine lees extracts, retention times (min), calibration curves, and coefficients of regression.

Compound	R_t_ (min)	Calibration Curve	R^2^
Petunidin 3-*O*-glucoside	3.565	y = 177800x − 3574.06	0.999
Peonidin 3-*O*-glucoside	3.899	y = 183944x − 3574.06	0.999
Malvidin 3-*O*-glucoside	4.402	y = 172752x − 3574.06	0.999
Cyanidin 3-(6″-acetylglucoside)	5.928	y = 173455x − 3574.06	0.999
Petunidin 3-(6″-acetylglucoside)	6.107	y = 163467x − 3574.06	0.999
Peonidin 3-(6″-acetylglucoside)	6.231	y = 168647x − 3574.06	0.999
Malvidin 3-(6″-acetylglucoside)	6.420	y = 159190x − 3574.06	0.999
Malvidin 3-(6″-*p*-caffeyglucoside)	6.542	y = 130025x − 3574.06	0.999
Cyanidin 3-(6″-*O*-*p*-coumaroyl-glucoside)	6.680	y = 143137x − 3574.06	0.999
Petunidin 3-(6″-*O*-*p*-coumaroyl-glucoside)	6.749	y = 136266x − 3574.06	0.999
Peonidin 3-(6″-*O*-*p*-coumaroyl-glucoside)	7.308	y = 133281x − 3574.06	0.999

**Table 4:** Results from the BBD–RSM analysis for total anthocyanin concentration and antioxidant activity of wine-lees extracts obtained by UAE.

Variables	Total Anthocyanins (mg/100 g dw)			DPPH (mg Trolox eq./g dw)		
	Sum of Squares	*F*-Value	*p*-Value	Sum of Squares	*F*-Value	*p*-Value
%EtOH	800.07	80.68	0.000	11.19	212.82	0.000
Temperature	7100.50	716.06	0.000	5.53	105.14	0.000
Cycle	0.00	0.00	0.985	0.00	0.03	0.874
Amplitude	5.32	0.54	0.470	0.00	0.06	0.805
pH	0.18	0.02	0.893	0.11	2.05	0.164
Ratio	11.87	1.20	0.284	0.08	1.44	0.240
%EtOH·%EtOH	913.50	92.12	0.000	12.03	228.64	0.000
%EtOH·Temperature	55.92	5.64	0.025	0.00	0.03	0.855
%EtOH·Cycle	1.26	0.13	0.724	0.00	0.01	0.939
%EtOH·Amplitude	6.97	0.70	0.410	0.00	0.06	0.804
%EtOH·pH	4.31	0.43	0.516	0.00	0.06	0.807
%EtOH·Ratio	0.34	0.03	0.855	0.00	0.03	0.855
Temperature·Temperature	13263.30	1337.56	0.000	85.12	1618.37	0.000
Temperature·Cycle	6.92	0.70	0.411	0.03	0.53	0.475
Temperature·Amplitude	0.90	0.09	0.766	0.15	2.93	0.099
Temperature·pH	1.42	0.14	0.708	0.00	0.06	0.804
Temperature·Ratio	2.12	0.21	0.648	0.09	1.80	0.191
Cycle·Cycle	1.49	0.15	0.702	0.38	7.31	0.012
Cycle·Amplitude	15.07	1.52	0.229	0.00	0.01	0.939
Cycle·pH	10.13	1.02	0.322	0.00	0.02	0.879
Cycle·Ratio	1.27	0.13	0.724	0.01	0.20	0.659
Amplitude·Amplitude	8.99	0.91	0.350	7.13	135.54	0.000
Amplitude·pH	5.95	0.60	0.446	0.00	0.03	0.867
Amplitude·Ratio	32.16	3.24	0.083	0.00	0.02	0.903
pH·pH	33.21	3.35	0.079	0.85	16.12	0.000
pH·Ratio	24.92	2.51	0.125	0.00	0.06	0.807
Ratio·Ratio	1.12	0.11	0.739	0.08	1.48	0.234
Total error	257.82			123.25		

**Table 5:** Repeatability and intermediate precision results for the total anthocyanin concentration (mg/100 g dw) and antioxidant activity of the extracts (mg TE/g dw) obtained under optimal conditions.

	Anthocyanins (mg/100 g dw)	Antioxidant Activity (mg TE/g dw)
*Repeatability*		
Average	150.25	8.06
SD *	1.10	0.19
RSD **	0.73	2.35
*Intermediate Precision*		
Average	148.88	7.88
SD *	1.37	0.26
RSD **	0.92	3.29

Repeatability (*n* = 9); Intermediate precision (*n* = 27); * Standard deviation; ** Relative standard deviation.

**Table 6:** Concentration of total anthocyanins (mg/100 g dw) and antioxidant activity (DPPH, mg TE/g dw) of Syrah and Cabernet Sauvignon wine lees.

Sample Code	Total Anthocyanins (mg/100 g dw)	Antioxidant Activity (mg TE/g dw)
Sy_MF_Ly	148.03 ± 1.71	7.82 ± 0.32
Sy_MF_Dr	112.62 ± 0.81	5.59 ± 0.42
CS_MF_Ly	81.17 ± 0.36	6.63 ± 0.10
CS_MF_Dr	53.50 ± 0.14	5.49 ± 0.78
CS_AF_Ly	81.99 ± 1.00	5.07 ± 0.01
CS_AF_Dr	27.72 ± 0.30	4.60 ± 0.00

## Data Availability

Data presented are contained within the article.
